# Simple surface functionalization of polymersomes using non-antibacterial peptide anchors

**DOI:** 10.1186/s12951-016-0205-x

**Published:** 2016-06-22

**Authors:** Ludwig Klermund, Sarah T. Poschenrieder, Kathrin Castiglione

**Affiliations:** Institute of Biochemical Engineering, Technical University of Munich, Boltzmannstraße 15, 85748 Garching, Germany

**Keywords:** Block copolymer, Hydrophobic peptide anchor, PMOXA-PDMS-PMOXA, Polymersomes, Surface functionalization, Non-antibacterial peptide

## Abstract

**Background:**

Hollow vesicles formed from block copolymers, so-called polymersomes, have been extensively studied in the last decade for their various applications in drug delivery, in diagnostics and as nanoreactors. The immobilization of proteins on the polymersomes’ surface can aid in cell targeting, lead to functional biosensors or add an additional reaction space for multistep syntheses. In almost all surface functionalization strategies to date, a chemical pre-conjugation of the polymer with a reactive group or ligand and the functionalization of the protein are required. To avoid chemical pre-conjugation, we investigated the simple and quick functionalization of preformed poly(2-methyloxazoline)-poly(dimethylsiloxane)-poly(2-methyloxazoline) (PMOXA-PDMS-PMOXA) polymersomes through the spontaneous insertion of four hydrophobic, non-antibacterial peptide anchors into the membrane to display enhanced green fluorescent protein (eGFP) on the polymersomes’ surface.

**Results:**

Three of the four hydrophobic peptides, the transmembrane domains of a eukaryotic cytochrome *b*_*5*_, of the viral lysis protein L and of the yeast syntaxin VAM3 could be recombinantly expressed as soluble eGFP-fusion proteins and spontaneously inserted into the polymeric membrane. Characterization of the surface functionalization revealed that peptide insertion was linearly dependent on the protein concentration and possible at a broad temperature range of 4–42 °C. Up to 2320 ± 280 eGFP molecules were immobilized on a single polymersome, which is in agreement with the calculated maximum loading capacity. The peptide insertion was stable without disrupting membrane integrity as shown in calcein leakage experiments and the functionalized polymersomes remained stable for at least 6 weeks.

**Conclusion:**

The surface functionalization of polymersomes with hydrophilic proteins can be mediated by several peptide anchors in a spontaneous process at extremely mild insertion conditions and without the need of pre-conjugating polymers.

**Electronic supplementary material:**

The online version of this article (doi:10.1186/s12951-016-0205-x) contains supplementary material, which is available to authorized users.

## Background

Block copolymers made of hydrophilic and hydrophobic blocks are capable of spontaneously forming hollow vesicles, so called polymersomes, when added to aqueous solutions. Due to their strong resemblance to liposomes [1] and their versatility, polymersomes have been studied for medical applications as drug delivery systems [2–4] or biosensors [5] or for biochemical applications as nano-scale membrane reactors [6, 7]. The vast variety of available polymers and the use of various different polymer chain lengths allow polymersomes to be tuned for desired characteristics. The ABA triblock copolymer poly(2-methyloxazoline)-poly(dimethylsiloxane)-poly(2-methyloxazoline) (PMOXA-PDMS-PMOXA) is a frequently used amphiphilic polymer in recent literature [8–11]. PMOXA-PDMS-PMOXA polymersomes show great stability and lower permeability than liposomes [1] while being biocompatible and low protein binding [9, 11, 12]. These characteristics allow retaining encapsulated drugs or enzymes within the vesicles, while the low protein binding properties are required to evade the immune system or prevent unspecific protein adsorption to the polymersomes’ surface.

The presentation of molecules on the surface is desired in all polymersome applications. In medical applications, surface functionalization can aid in the specific targeting, cellular uptake or controlled degradation of the polymersomes, thus allowing a controllable distribution within the body [3]. In biochemical applications, surface functionalization can make the outer reaction space available for compartmentalized multistep syntheses, which may be not or only partially compatible, while retaining the possibility to recover the catalytic species as a whole entity [7].

So far, numerous approaches have been pursued to functionalize the surface of polymer membranes, indicating the need for quick and simple strategies to immobilize proteins on polymersome surfaces. Focus has been laid on chemical conjugation [6, 7, 13–16] and on non-covalent binding using the interaction of biotin and streptavidin [9, 17–19]. These methods require the pre-conjugation of the polymer and the protein with reactive groups, e.g., azides and alkynes, or interaction partners, e.g., biotin and streptavidin, thereby adding multiple additional steps to the surface functionalization of polymersomes.

The polymersomes’ resemblance to liposomes allows biological transmembrane domains to span the inner membrane. The general feasibility of spontaneously inserting proteins into PMOXA-PDMS-PMOXA polymersomes has already been demonstrated for various transmembrane channels [11, 17, 20–26] and antibacterial, pore forming peptides [5, 26–29]. Recently, Noor et al. have presented the immobilization of enhanced green fluorescent protein (eGFP) on the surface of poly(isobutylene)-poly(ethylene glycol)-poly(isobutylene) (PIB-PEG-PIB) polymersomes with the antibacterial peptide cecropin A (CecA) [30]. This strategy offers a strong interaction between the polymer and the protein at mild conditions and is not constrained to pre-conjugated polymers [30].

Because the polymersomes are to be used as drug carriers, in diagnostics or as bioreactors, uncontrolled diffusion across the polymer membrane may be detrimental, making antibacterial peptides not applicable for surface functionalization due to their potential to disrupt membrane integrity and cause leakage of entrapped molecules. Although Noor et al. stress that CecA does not form pores in PIB-PEG-PIB polymersomes, other antibacterial peptides such as alamethicin [26] and gramicidin [5] readily insert into and destabilize PMOXA_13_-PDMS_33_-PMOXA_13_ and PMOXA_7_-PDMS_60_-PMOXA_7_ membranes, respectively, suggesting sufficient membrane compressibility and fluidity as well as peptide solvation for pore assembly [8, 10, 24, 30, 31].

Natural peptide anchors exist which tether their adjacent protein or enzyme to membranes but do not form pores or disintegrate lipid membranes. These peptide anchors, of which the most common member is the C-terminal domain of the cytochrome *b*_*5*_, do not require a signal recognition particle or translocation apparatus for membrane insertion, but insert into preformed lipid bilayers spontaneously [32].

To avoid chemical conjugation and circumvent problems with membrane integrity, we focused on the functionalization of PMOXA_15_-PDMS_68_-PMOXA_15_ polymersomes with four peptide anchors that differ in length and hydrophobicity and are not expected to destabilize or rupture biological membranes. The four peptide anchors comprised the transmembrane domain of the rabbit cytochrome *b*_*5*_ (Cyt*b*_*5*_′) [33–35], the transmembrane domain of the viral lysis protein L (L′) of the bacteriophage MS2 [36, 37], the transmembrane domain of the yeast syntaxin VAM3 (Vam3p′) [38] and an artificial peptide consisting of an alpha-helical repetition of alanines and leucines (PolyAL) [39]. In this study, we demonstrate that the surface functionalization of polymer membranes is not limited to antibacterial peptides and integral membrane proteins, but can be performed with a variety of natural peptide anchors which are capable of immobilizing proteins on the polymersome surface and are especially suitable for the functionalization of polymersomes for medical and biotechnological applications.

## Results

**Fig. 1:**
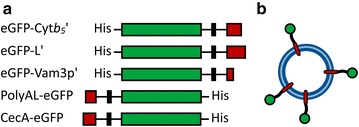
Schematic depiction of eGFP-fused peptide anchors and functionalized polymersomes. **a** Fusion proteins with eGFP (*green*), the respective peptide anchor (*red*) and a decaalanine linker (*black*). The hexahistidine tag (His_6_) was cloned opposite of the peptide anchor to not interfere with peptide insertion. **b** Functionalized polymersomes with the same *color* code

For the determination of the insertion behavior of the non-antibacterial peptide anchors Cyt*b*_*5*_′, L′, Vam3p′ and PolyAL and their ability to immobilize protein on PMOXA_15_-PDMS_68_-PMOXA_15_ membranes, each peptide anchor was genetically fused to eGFP. This allowed for an easy detection and quantification of the insertion via fluorescence intensity. C*ytb*_*5*_′, *l′* and *vam3p*′ were cloned C-terminal of *egfp* while *polyAL* was cloned N-terminal of *egfp*, resulting in the fusion proteins eGFP-Cyt*b*_*5*_′, eGFP-L′, eGFP-Vam3p′ and PolyAL-eGFP (Fig. 1). As a reference peptide, the gene of the antibacterial CecA was cloned N-terminal of *egfp* (CecA-eGFP) according to Noor et al. [30]. The properties of the peptide anchors are summarized in Table 1. With a content of hydrophobic amino acids of 96 %, PolyAL represents the most hydrophobic peptide anchor, followed by Vam3p′ with 84 % and Cyt*b*_*5*_′, L′ and CecA′ with approximately 50 % each. The peptide lengths range from 19 to 55 amino acids.

**Table 1:** Properties of the peptide anchors

	Cyt*b*_*5*_′	L′	Vam3p′	PolyAL	CecA
Length, # amino acids	43	55	19	31	37
Hydrophobicity, % hydrophobic amino acids	56	47	84	96	46
Isolated yield, mg L^−1^	2.7	5.5	2.1	n/a	1.2
Relative fluorescence, %	103 ± 6	103 ± 10	27 ± 6	n/a	38 ± 4
Inserted molecules per polymersome per µM initial protein^a^	20.7 ± 0.7	4.8 ± 0.2	3.7 ± 0.4	n/a	1.6 ± 0.1

*n/a* not available

^a^At 37 °C for 4 h with 0.5 % w/v polymersomes

### Expression and purification

The fusion proteins eGFP-Cyt*b*_*5*_′, eGFP-L′, eGFP-Vam3p′, PolyAL-eGFP and CecA-eGFP were recombinantly expressed in *E. coli* BL21(DE3). Because the hydrophobic peptide anchors were expected to insert and tether the protein to the cytosolic membrane after expression, an additional membrane solubilization step was introduced after cell lysis to solubilize the membrane-bound protein. With the detergent nonident P-40 (NP-40), the target protein yield could be increased up to sixfold, resulting in isolated yields of 2.7 mg eGFP-Cyt*b*_*5*_′, 5.5 mg eGFP-L′ and 2.1 mg eGFP-Vam3p′ per liter expression culture (Table 1). Isolated yields of CecA-eGFP were 1.2 mg per liter expression culture.

Because NP-40 strongly affects the membrane integrity of PMOXA_15_-PDMS_68_-PMOXA_15_ polymersomes (Additional file 1: Fig. S1), no detergent was used during the purification steps to reduce residual detergent to a minimum. Typical protein concentrations after purification ranged from 50 to 500 µg mL^−1^. Interestingly, high salt concentrations (500 mM NaCl, 270 mM imidazole) stabilized the hydrophobic peptide anchors in solution and prevented protein precipitation during storage. Especially eGFP-Vam3p′ quickly precipitated when dialyzed against low salt buffers (0.1 M Tris–HCl, pH 8.0). Except for eGFP-Vam3p′, the fusion proteins were stable for weeks at protein concentrations of up to 500 µg mL^−1^ when stored in high salt buffers, making Cyt*b*_*5*_′, L′ and CecA most suitable for application in terms of storage stability.

No detectable fluorescence was measured when expressing PolyAL-eGFP, indicating that the extremely hydrophobic PolyAL-eGFP was not expressed in functional form. SDS-PAGE revealed that small amounts of PolyAL-eGFP were present in the insoluble cell debris, however, treatment with NP-40 led to no increase in fluorescence or protein yield. Since we observed a solubilization of the other peptide anchors upon NP-40 treatment, the low protein yield suggests that the hydrophobic peptide PolyAL is not inserted into the membrane but strongly aggregates to inclusion bodies. To prevent the peptide anchor from aggregating, or inserting into the membrane, the solubility-enhancing maltose binding protein (MBP), including a tobacco etch virus (TEV) protease cleavage site, was cloned N-terminal of PolyAL-eGFP, leading to a cleavable MBP-TEV-PolyAL-eGFP fusion protein (Additional file 1). Unfortunately, the MBP-TEV moiety did not render the protein more soluble as judged by SDS-PAGE and fluorescence measurements. Thus, due to its strong hydrophobicity, PolyAL could not be expressed in soluble form and was therefore not further considered a suitable peptide anchor for the spontaneous insertion into polymer membranes and no further attempts to express the protein were performed.

### Influence on eGFP fluorescence

To assess the influence of the peptide anchors on the correct folding of eGFP, the fluorescence intensity of each purified fusion protein was compared to eGFP without peptide anchor (Table 1). EGFP retained full fluorescence when fused to Cyt*b*_*5*_′ and L′ with 103 ± 6 and 103 ± 10 % relative fluorescence. CecA-eGFP and eGFP-Vam3p′ had relative fluorescence intensities of 38 ± 4 and 27 ± 6 %, respectively, indicating a negative effect of CecA and Vam3p′ on protein folding and chromophore formation.

### Peptide insertion into polymersomes

**Fig. 2:**
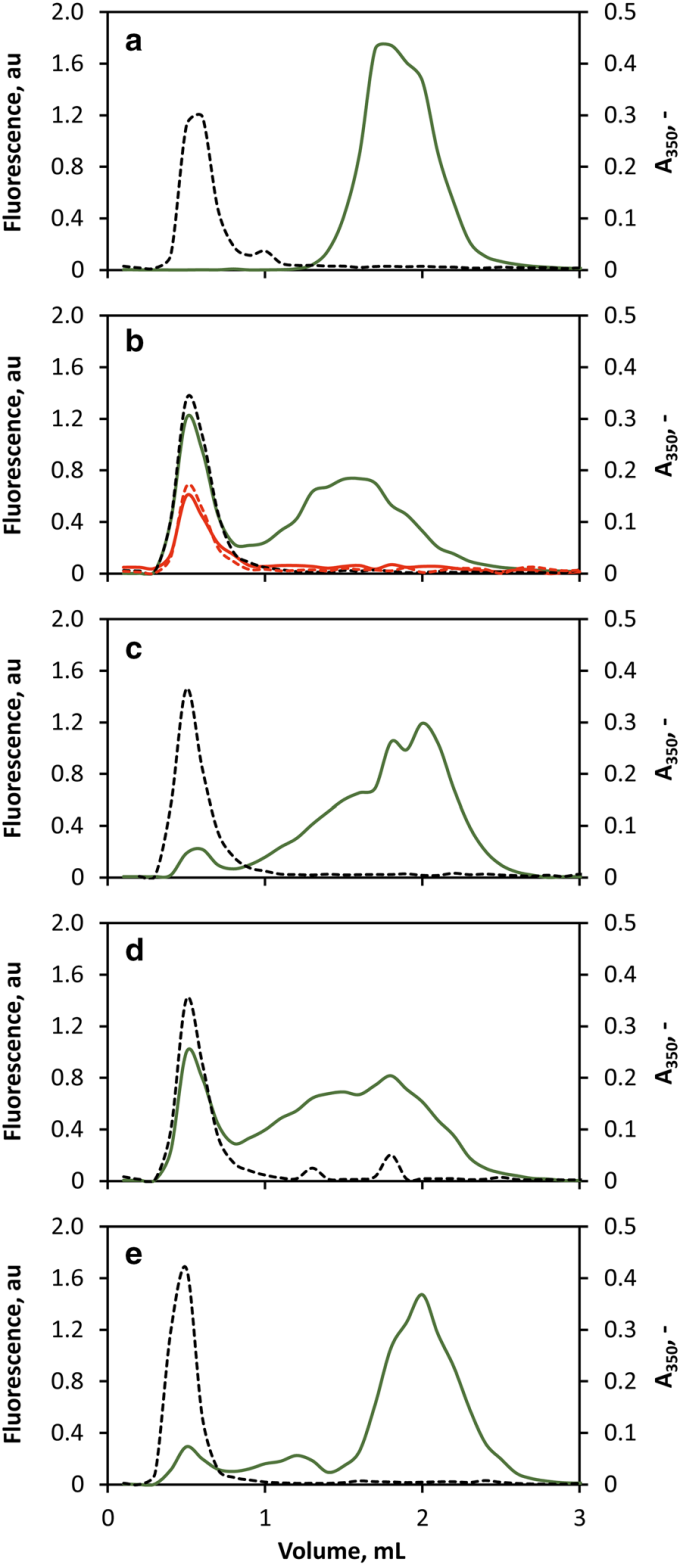
Size-exclusion chromatography of PMOXA_15_-PDMS_68_-PMOXA_15_ polymersomes. **a** eGFP, **b** eGFP-Cyt*b*_*5*_′, **c** eGFP-L′, **d** eGFP-Vam3p′ and **e** CecA-eGFP after surface functionalization for 4 h at 37 °C. Polymersomes were quantified by measuring light absorbance at 350 nm (*black, dashed*), the presence of eGFP was verified by fluorescence signal (*green, solid*). A re-loading of functionalized polymersomes after 6 weeks of storage is exemplarily shown for eGFP-Cyt*b*_*5*_′ (*red*). Each run is the mean of a triplicate determination

Since adding fusion protein during the polymersome formation would presumably lead to a nondirectional integration of the peptide anchor, yielding eGFP on the outer and the inner surface of the membrane, the insertion of the peptide anchors into PMOXA_15_-PDMS_68_-PMOXA_15_ membranes was studied by adding CecA-eGFP, eGFP-Cyt*b*_*5*_′, eGFP-L′ and eGFP-Vam3p′ to preformed polymersomes. PMOXA_15_-PDMS_68_-PMOXA_15_ polymersomes were thus formed by self-assembly in 12 mL-stirred tank reactors under vigorous stirring according to the method of Poschenrieder et al. until a particle size distribution with a polydispersity index below 0.2 was reached [40]. After addition of the fusion proteins to the preformed polymersomes and subsequent incubation for 4 h at 37 °C, functionalized polymersomes were separated from free protein by size-exclusion chromatography (SEC). The polymersomes were detected by measuring the absorbance at 350 nm, which resulted in a defined polymersome peak at a retention volume of 0.6 mL (Fig. 2, black, dashed), whereas eGFP was detected by measuring the fluorescence intensity (green, solid). Without peptide anchor, eGFP was separated from the polymersomes at high resolution, indicating no unspecific interactions between the eGFP moiety and the polymersomes (Fig. 2a). Figure 2b–e depict the absorbance and fluorescence chromatograms of polymersomes functionalized with eGFP-Cyt*b*_*5*_′, eGFP-L′, eGFP-Vam3p′ and CecA-eGFP, respectively. Successful insertion of the peptide anchors into the polymer membrane was detected for each peptide anchor by measuring fluorescence in the fractions containing polymersomes and thus a co-localization of the absorbance peak and the fluorescence peak, demonstrating that each peptide anchor is capable of tethering eGFP to the polymersomes’ surface. The lack of interaction between the eGFP moiety and the polymersomes validates that adhesion of eGFP to the polymersomes is indeed conferred by the peptide anchors.

Re-loading of functionalized polymersomes onto SEC columns, as exemplarily shown for eGFP-Cyt*b*_*5*_′ in Fig. 2b (red), led to a single fluorescence peak co-localized with the absorbance peak of the polymersomes without the presence of free eGFP-Cyt*b*_*5*_′. The decrease in fluorescence and absorbance results from a reduced sample loading due to dilution effects of the SEC. No dissociation of the hydrophobic peptide anchors from the membrane was observed within the measured time span of 6 weeks of storage, which clearly demonstrates a strong hydrophobic interaction between the peptide anchors and the membrane core and a high stability of immobilized eGFP.

**Fig. 3:**
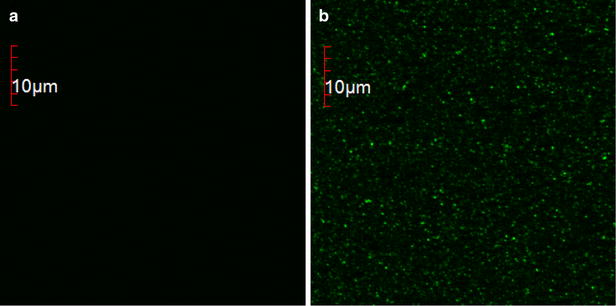
Confocal microscopy images. **a** Non-functionalized polymersomes. **b** Purified, eGFP-functionalized polymersomes using Cyt*b*_*5*_′ as peptide anchor. The *scale bar* is 10 µm

To visualize the adhesion of eGFP to the polymersomes, confocal microscopy images of polymersomes before and after functionalization with peptide anchors were taken. A clustering of the fluorescence to the polymersomes was observed after functionalization with peptide anchor-fused eGFP only, further demonstrating peptide insertion and eGFP immobilization on the polymersome surfaces (Fig. 3).

Qualitative comparison of the peptide anchors revealed that approximately 26 % of the total amount of eGFP-Cyt*b*_*5*_′ inserted into the polymersomes. Employing Vam3p′ as peptide anchor, approximately 10 % of the fluorescence was measured in the fractions containing polymersomes, whereas for CecA-eGFP and eGFP-L′, approximately 6 % of the total fluorescence were co-localized with the polymersomes, indicating that a majority of the protein remains in solution. The data suggest that a certain hydrophobicity of the peptide anchor is required for spontaneous peptide insertion, as CecA and L′ are the least hydrophobic peptides. Furthermore, peptide length may influence the peptide insertion efficiency due to mismatch phenomena. This mismatch between peptide length and membrane thickness arises from the larger thickness of the polymer membrane of approximately 10–15 nm compared to the thickness of natural lipid bilayers of 5–6 nm. Although it has been demonstrated that polymer membranes are highly compressible [10, 31], naturally occurring membrane peptides and integral membrane proteins are evolutionarily optimized to span natural bilayers, which in turn may impose a negative effect on peptide insertion into the polymer membrane [31]. However, the length of the peptide anchors had no clear effect on membrane insertion, despite covering a broad range of 19–55 amino acids. Each peptide anchor was capable of anchoring eGFP to the polymersomes’ surface, regardless of the differences in hydrophobicity and peptide length, establishing a general feasibility of spontaneously inserting non-antibacterial peptide anchors into polymer membranes and suggesting that other non-antibacterial peptides, which have not been covered in this study, also integrate into polymer membranes.

### Insertion behavior of peptide anchors

**Fig. 4:**
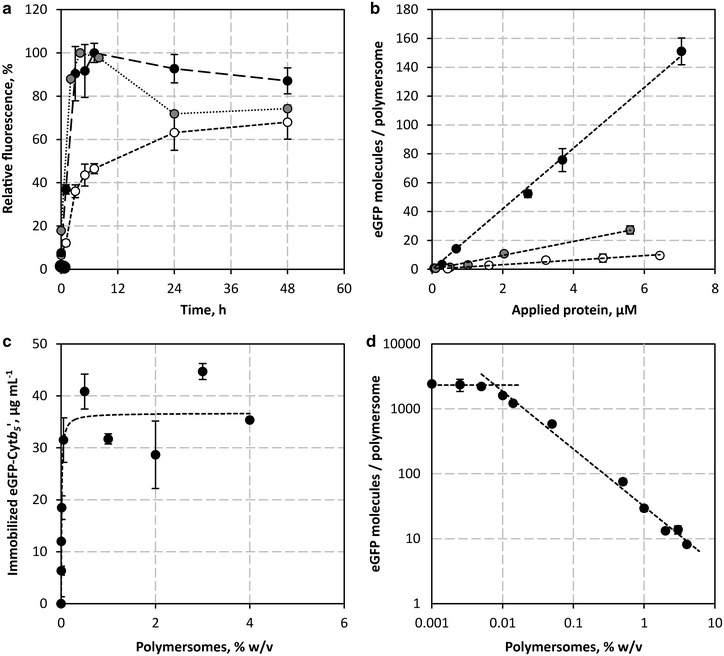
Insertion behavior of peptide anchors. The presented values are the mean ± SD of at least three experiments. **a** Relative insertion of eGFP-Cyt*b*_*5*_′ into preformed PMOXA_15_-PDMS_68_-PMOXA_15_ polymersomes at 4 °C (*white*), 37 °C (*black*) and 42 °C (*gray*). **b** Concentration dependent peptide insertion into 0.5 % w/v PMOXA_15_-PDMS_68_-PMOXA_15_ polymersomes after 4 h at 37 °C, exemplarily shown for eGFP-Cyt*b*_*5*_′ (*black*), eGFP-L′ (*gray*) and CecA-eGFP (*white*). **c** Dependence of the amount of immobilized eGFP on the polymersome concentration, exemplarily shown for an initially applied 130 µg mL^−1^ eGFP-Cyt*b*_*5*_′. Insertion follows saturation kinetics (*dashed line*). **d** Log–log plot of eGFP-Cyt*b*_*5*_′ molecules per polymersome that can be inserted at various polymersome concentrations

A detailed characterization of the insertion behavior revealed that the peptide anchors inserted spontaneously into the polymer membrane at temperatures ranging from 4 to 42 °C, as exemplarily shown in Fig. 4a for eGFP-Cyt*b*_*5*_′. Cyt*b*_*5*_′ and L′ showed maximum insertion into the polymersomes within 3–7 h at 37 and 42 °C. Incubation for more than 12 h led to a decrease in fluorescence, which is more prominent at 42 °C and may be due to heat denaturation of eGFP. At 4 °C, maximum insertion was reached after approximately 48 h. Prolonged incubation led to no further insertion, indicating that peptide insertion is equilibrium controlled. In contrast Vam3p′ showed a strongly reduced insertion at 37 °C, which was mainly due to instability of the fusion protein in solution above room temperature. With a glass transition temperature of the PDMS block of −123 °C [41], no glass transition of the hydrophobic block occurs between 4 and 42 °C and we expect that the increase in the time required for insertion at lower temperatures is mainly due to reduced Brownian motion rather than a more condensed membrane structure.

To obtain a quantitative measure of the peptide insertion, the amount of immobilized eGFP was quantified from the obtained fluorescence intensities using linear standards (Additional file 1: Figs. S2–S4). The number of polymersomes per liter was estimated from the mass percent concentration of the polymersome dispersion, the molar mass of the copolymer and the average aggregation number of 43,000 polymers per polymersome [40]. At a constant polymersome concentration, a linear increase of immobilized eGFP per polymersome was observed with increasing protein concentration (Fig. 4b). At 0.5 % w/v polymersomes, eGFP-Cyt*b*_*5*_′ showed the highest insertion efficiency of 20.7 ± 0.7 eGFP-Cyt*b*_*5*_′ molecules per polymersome per micromolar applied protein, followed by eGFP-L′, eGFP-Vam3p′ and CecA-eGFP with insertion efficiencies of 4.8 ± 0.2, 3.7 ± 0.4 and 1.6 ± 0.1 molecules per polymersome per micromolar applied protein, respectively. Thus, by doubling the protein concentration, double the amount of eGFP was immobilized on the polymersomes’ surface. This implies a constant ratio between immobilized and free protein. Re-applying non-bound protein to unloaded polymersomes led to the same ratio of immobilized to free protein. Similarly, applying protein to already functionalized polymersomes also led to the same ratio between immobilized and free protein, demonstrating an equilibrium controlled insertion. However, desorption of inserted peptide anchors was not observed when applying functionalized polymersomes to a protein-free solution.

At the highest protein concentration of 7.1 µM eGFP-Cyt*b*_*5*_′, Cyt*b*_*5*_′ immobilized approximately 150 eGFP molecules per polymersome with a total of 9.2 × 10^15^ polymersomes per litre (0.5 % w/v), whereas up to 27 eGFP-L′, 16 eGFP-Vam3p′ and 10 CecA-eGFP molecules were immobilized per polymersome at slightly lower initial protein concentrations of 5.6 µM eGFP-L′, 4.6 µM eGFP-Vam3p′ and 6.4 µM CecA-eGFP.

**Fig. 5:**
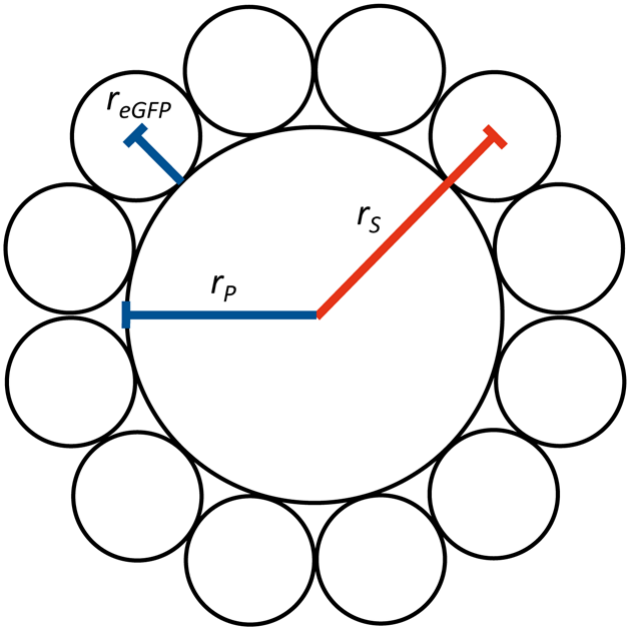
Schematic depiction of the radii used for the calculation of the theoretical maximum loading capacity of polymersomes. The radius *r*_*S*_ was used for calculating the available surface area with *r*_*S*_ = *r*_*P*_ + *r*_*eGFP*_, where *r*_*P*_ is the radius of the polymersome and *r*_*eGFP*_ is the hydrodynamic radius of eGFP

In contrast to changing the applied protein concentration, increasing or decreasing the polymersome concentration, which is equivalent to a change in the available surface area for peptide insertion, had no effect on the absolute amount of eGFP that was immobilized at a constant applied protein concentration. The absolute amount of immobilized eGFP per polymersome concentration followed an apparent saturation kinetics with a maximum of 38 µg mL^−1^ immobilized eGFP-Cyt*b*_*5*_′ at an initially applied 130 µg mL^−1^ eGFP-Cyt*b*_*5*_′ (Fig. 4c). Thus, peptide insertion seems to be controlled by an equilibrium between immobilized and free protein independent of the available surface area. In log–log scale (Fig. 4d) this behavior is represented by an inversely proportional dependency of the immobilized eGFP molecules per polymersome with increasing polymersome concentration. Below 0.05 % w/v, the polymersome surface area becomes limiting, resulting in a constant amount of immobilized eGFP molecules per polymersome of 2320 ± 280. This value is in perfect agreement with the theoretical maximum loading capacity of 2254 eGFP molecules that can be presented on a single polymersome without steric hindrance (Fig. 5). The maximal loading capacity was calculated from the available surface area *A*_*S*_ of a single polymersome (Eq. 1) and the largest two-dimensional area *A*_*eGFP*_ that is occupied by each eGFP on the surface, which can be calculated from any plane going through the center of a globular protein according to Eq. 2.

1 $$A_{S} = 4\pi \cdot r_{S}^{2}$$

2 $$A_{eGFP} = \pi \cdot r_{eGFP}^{2}$$

For the case of the eGFP molecules lying directly on the polymersome’s surface, the radius *r*_*S*_ determines the available surface area and must be extended by the distance from the polymersome’s surface to the protein core according to Eq. 3.

3 $$r_{S} = r_{P} + r_{eGFP} = \frac{{d_{P} }}{2} + r_{eGFP}$$

Thus, the radius *r*_*S*_ was derived from the number-based mean diameter of the polymersomes *d*_*P*_ of 110 nm, which was measured by dynamic light scattering, and the hydrodynamic radius *r*_*eGFP*_ of eGFP of 2.3 nm [42]. At the highest-density hexagonal packing arrangement of equal circles, 90.7 % of the available surface area *A*_*S*_ (90.7 % *A*_*S*_ = 37,422.8 nm^2^) can be covered by the circular areas occupied by eGFP (*A*_*eGFP*_ = 16.6 nm^2^), resulting in a maximum loading capacity of 2254 eGFP molecules per polymersome.

### Membrane integrity during peptide insertion

**Fig. 6:**
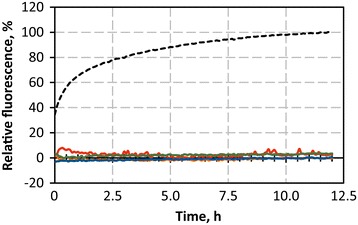
Calcein leakage experiments. Calcein release during insertion of Cyt*b*_*5*_′ (*red*), L′ (*orange*), Vam3p′ (*green*) and CecA (*blue*) into calcein-loaded polymersomes. A reduction in membrane integrity leads to the release of self-quenched calcein from the polymersome interior and a subsequent increase in fluorescence and was only observed for the positive control, which was treated with 3 % triton X-100 to rupture the polymer membrane (*black, dashed*)

Because membrane integrity is crucial for polymersome applications, calcein leakage experiments were performed to investigate membrane integrity during peptide anchor insertion. Polymersomes were loaded with calcein at a self-quenching concentration and the calcein release during peptide insertion was monitored via an increase in fluorescence. As expected, no calcein leakage was detected within 12 h of incubation of each peptide anchor with calcein-loaded polymersomes at 37 °C (Fig. 6). Thus, none of the peptides substantially destabilized or formed pores within the polymer membrane. Notably, the insertion of CecA, which is known to form pores in lipid membranes, led to no calcein leakage when added at a peptide-to-polymer ratio of 1:200. Although CecA-induced calcein release is concentration dependent, calcein release from liposomes has been demonstrated for peptide-to-lipid ratios of 1:280 and lower [43], indicating that either polymersomes are more stable than liposomes, pore formation is sterically hampered by the hydrophilic eGFP moiety or CecA insertion is not as efficient as in lipid membranes.

Because membrane integrity is of high priority when it comes to the use of polymersomes in medical or biochemical applications, membrane integrity during the functionalization of the polymersome surface is of essential importance. With Cyt*b*_*5*_′, L′ and Vam3p′, we identified three peptide anchors that are not known to form pores in biological membranes and did not form pores in polymersomes.

## Discussion

In this study, we demonstrated that the hydrophobic, non-antibacterial peptide anchors Cyt*b*_*5*_′, L′ and Vam3p′ readily insert into preformed PMOXA_15_-PDMS_68_-PMOXA_15_ polymersomes in a similar manner to the various membrane proteins [20, 22, 23, 25, 26] and antibacterial peptides [26, 27, 29, 30] that have been incorporated into polymer membranes in the recent years. Peptide insertion occurred immediately after addition of the peptide anchors to the polymer vesicles, which is evidence that the hydrophobic driving force of non-antibacterial peptides is strong enough to penetrate and insert into polymeric membranes despite being soluble in aqueous solution.

Remarkably, the insertion kinetics of the peptide anchor Cyt*b*_*5*_′ into polymersomes are strikingly similar to full length Cyt*b*_*5*_ insertion into liposomes despite the differences in membrane composition and thickness. Rogers and Strittmatter obtained a maximum Cyt*b*_*5*_ insertion within 50 h at 4 °C and within 10 h at 32 °C [34] which is in congruence with a maximum insertion obtained after 48 h at 4 °C and within 7 h at 37 °C for peptide anchor insertion into polymersomes. The similar behavior of polymersomal and liposomal integration of hydrophobic peptides is supported by a recent study by Itel et al. who report that the lateral diffusion of proteins within polymer membranes is intriguingly similar to lipid membranes and that the polymer compressibility allows the incorporation of hydrophobic peptides, regardless of size and structure [10].

Furthermore, a concentration dependent increase in peptide insertion for Cyt*b*_*5*_ into liposomes with a maximum loading capacity of 244 molecules per liposome was observed, which is equivalent to a peptide-to-lipid ratio of 1:11 [34]. Using polymer vesicles, we were able to achieve a similar insertion with a peptide-to-polymer ratio of 1:17 at the maximum loading capacity of 2320 ± 280 molecules per polymersome. With an average number-based mean diameter of 110 nm of the polymersomes and a hydrodynamic radius of eGFP of 2.3 nm, a theoretical 2254 molecules can be presented on a single polymersome at the highest-density hexagonal packing arrangement of equal circles, thus validating that the results are in good agreement with the theoretical maximum degree of functionalization per polymersome.

For the potential use of polymersomes in medical and biotechnological applications, the surface functionalization of polymer membranes plays a major role, which is reflected in the many different strategies that have been proposed for immobilizing proteins and other ligands on polymer surfaces. These include non-covalent interactions of biotinylated polymers and ligands [44], non-covalent interactions of Ni^2+^-NTA-conjugated polymers with His_6_-tagged proteins [45], chemical conjugation using click-chemistry [15], and genetic fusion of antibacterial peptides [30] to the target protein.

Although several immobilization strategies have been investigated, only few data exist that allow for a quantitative comparison. Noor et al., who have investigated the immobilization of eGFP using the antibacterial peptide CecA, unfortunately give no quantified data on the immobilization events. However, the non-covalent immobilization employing Ni^2+^-NTA-functionalized PB-PEO polymersomes report a maximum of 24 immobilized red fluorescent protein molecules per polymersome [45], which is in range with our measurements with 0.5 % w/v polymersomes harboring up to 16–150 eGFP molecules depending on the peptide anchor. With a maximum loading capacity of 2320 ± 280 eGFP molecules that could be immobilized using the peptide anchor Cyt*b*_*5*_′ per single polymersome, the use of non-antibacterial peptides for surface functionalization reaches similar values as the maximum loading capacity of biotinylated PMOXA-PDMS diblock polymersomes of 1921 ± 357 fluorescently labled avidin molecules [44]. In contrast, Egli et al. have reported the immobilization of 57 molecules of the fluorescent dye Alexa Fluor 633 but only five enhanced yellow fluorescent protein molecules per polymersome using chemical conjugation of 4-formylbenzoate-conjugated PMOXA-PDMS diblock polymersomes to 6-hydrazinonicotinate acetone hydrazine, suggesting that the accessibility of the pre-conjugated polymer may be a problem when immobilizing proteins compared to significantly smaller fluorescent dyes.

Thus, the surface functionalization of polymersomes with hydrophobic, non-pore forming peptide anchors performs extremely well compared to other, more complex immobilization techniques. The simplicity of the protein immobilization elevates the surface functionalization using hydrophobic peptide anchors to an easy to use, versatile and simple surface functionalization tool that can be performed at extremely mild conditions (e.g., 4 °C, neutral pH) and does not require tedious chemical pre-conjugation of the polymer.

The main challenge for using peptide anchors to immobilize target proteins on polymer surfaces is not the insertion of the peptide into the membrane but finding a peptide anchor with sufficient amphiphilicity to be recombinantly expressible in soluble form, have little effect on the target protein and exhibit enough hydrophobic force to spontaneously insert into the membrane. With Cyt*b*_*5*_′ and L′, we identified two peptide anchors that readily insert into PMOXA_15_-PDMS_68_-PMOXA_15_ polymersomes, have no effect on eGFP fluorescence and are stable in aqueous solution after recombinant expression in *E. coli*. The transferability to other proteins or enzymes is currently under investigation to extend the scope of possible applications.

## Conclusion

In this study we show that the surface functionalization of polymersomes with a hydrophilic protein can be mediated by several hydrophobic peptide anchors and is not limited to antibacterial peptides or pre-conjugated polymers. The purified eGFP-coupled peptide anchors Cyt*b*_*5*_′, L′ and Vam3p′ spontaneously inserted into PMOXA_15_-PDMS_68_-PMOXA_15_ membranes in a concentration dependent manner at a broad temperature range from 4 to 42 °C. Up to 2320 ± 280 eGFP-Cyt*b*_*5*_′ molecules were immobilized per polymersome with an average of 9.2 × 10^13^ polymersomes per litre. This exceeds the largest amount of protein that has been immobilized on a single polymersome found in literature by a 100-fold. Furthermore, the peptide insertion is not limited to antibacterial peptides, which are amphiphilic by nature to be both soluble in aqueous solution and to insert spontaneously into membranes, but could be performed with eukaryotic and viral peptides that are not capable of pore formation. Since the capability of pore formation is an intrinsic property of the peptide, no pore formation is expected for Cyt*b*_*5*_′, L′ and Vam3p′ in any polymeric membrane, regardless of the peptide concentration and the thickness, compressibility and fluidity of the membrane, making these peptide anchors universally applicable for any type of polymersome.

## Methods

### Materials

PMOXA_15_-PDMS_68_-PMOXA_15_ (M_W_/M_N_ = 1.23; M_W_: mass-average molecular mass; M_N_: number-average molecular mass) was purchased from Polymer Source Inc. (Dorval, Canada). Enzymes used for DNA work were obtained from New England Biolabs (Frankfurt, Germany). Oligonucleotides were purchased from biomers.net (Ulm, Germany) and Eurofins Genomics (Ebersberg, Germany). All other chemicals were of analytical grade from various suppliers.

### Molecular cloning

Details on the DNA sequences encoding the peptide anchors and the cloning procedures are given in the supplementary information (Additional file 1: Table S1). In short, the *egfp* gene was cloned into pET28a(+) and pET21a(+) vectors (Novagen, Madison, USA). The genes coding for *cytb*_*5*_*′*, *l*′ and *vam3p′* were inserted C-terminal to the *egfp* gene into pET28a(+)-eGFP. *Ceca* and *polyAL* were inserted N-terminal to the *egfp* gene into pET21a(+)-eGFP. The *egfp* gene and the respective anchor sequence were separated by an oligonucleotide encoding a decaalanine linker. The pET28a(+) vector constructs included an N-terminal, the pET21a(+) vector constructs included a C-terminal hexahistidine (His_6_)-tag in frame with the cloned genes. All plasmids were sequenced by Eurofins Genomics (Ebersberg, Germany) and transformed into *E. coli* BL21(DE3) (Novagen, Madison, USA) for protein expression.

### Protein expression and purification

Protein expression was performed as described previously [46] with the sole exception of using terrific broth during expression. The cells were harvested by centrifugation and resuspended in 5 mL binding buffer (20 mM sodium phosphate buffer, pH 7.4, 500 mM NaCl, 40 mM imidazole) per gram cell wet weight. The cells were lysed by sonication (245–260 µm amplitude, 0.5 s pulses) (Sonoplus HD 2070 equipped with sonotrode MS73; Bandelin, Berlin, Germany) for 3 min and the lysate was solubilized in 2 % v/v NP-40 for 1 h at room temperature in a rotary shaker. After centrifugation (50,000×*g*, 30 min), the fusion protein was purified using a 1-mL HisTrap FF column (GE Healthcare, Uppsala, Sweden) according to Groher and Hoelsch [47]. The collected protein was polished using a HiTrap Butyl FF column (GE Healthcare). The column was equilibrated with 3 M NaCl. Protein was applied in the elution buffer (20 mM phosphate buffer, pH 7.4, 0.5 M NaCl, 270 mM imidazole) and the column was washed with 10 mL washing buffer (40 mM phosphate buffer, pH 7.4). Anchor peptides fused to eGFP were eluted with bidistilled water and dialyzed against elution buffer. CecA-eGFP was washed with 1 M NaCl and eluted in elution buffer. Protein purity was judged by SDS-PAGE stained with Coomassie. Protein concentrations were determined with the bicinchoninic acid assay (Pierce, Rockford, USA) using bovine serum albumin as standard.

### Polymersome preparation

PMOXA_15_-PDMS_68_-PMOXA_15_ was solubilized in ethanol (99.8 %) to obtain a 20 % w/v polymer solution. The polymer solution was added to deionized water in a 1:20 ratio to obtain a 1 % w/v polymersome dispersion and stirred in 12 mL-stirred tank reactors at 4000 rpm with an S-type stirrer for 1.25 h according to the method of Poschenrieder et al. [40]. The polydispersity index, the intensity-based diameter (z-average) and the number-based mean diameter (*d*_*P*_) of the polymersomes were measured via dynamic light scattering on a ZetaSizer Nano-S (Malvern Instruments, Malvern, UK). For each polymersome preparation, the polydispersity index was ≤0.2 with a z-average of approximately 185 nm in a narrow, monomodal size distribution and a *d*_*P*_ of 110 nm. The average aggregation number has been determined to 43,000 polymer chains per polymersome [40]. Polymersome concentrations above 1 % w/v were obtained by concentrating the polymersome dispersion in 10 kDa Vivaspin 20 ultrafiltration units (Sartorius, Göttingen, Germany).

### Peptide anchor insertion

Peptide insertion was performed at various temperatures in 10 mM phosphate buffer, pH 7.4, 250 mM NaCl and 135 mM imidazole at 600 rpm in a thermal shaker. The protein concentration was varied from 5 to 250 µg mL^−1^, the polymersome concentration was varied from 0.001 to 4 % w/v.

### Size-exclusion chromatography

Immobilized protein was separated from free protein by size exclusion chromatography. Samples (0.1 mL, 4 % of total bed volume) were applied on 2.5 mL laboratory columns (MoBiTec, Göttingen, Germany). 3.7 mL running buffer (0.1 M Tris–HCl, pH 8.14) were added in 100 μL increments, allowed to pass through the column by gravitational force, and collected in microcentrifuge tubes. The eGFP and polymersome concentration was determined in each fraction.

### Absorbance and fluorescence measurements

Polymersomes were detected and quantified by absorbance at 350 nm. The fractions that contained polymersomes were qualitatively analyzed via dynamic light scattering.

EGFP fluorescence was measured with an Infinite M200 microplate reader (Tecan, Männedorf, Switzerland) using 96 well, half area, black polystyrene assay plates (Corning Inc., Corning, USA) and excitation and emission wavelengths of 485 and 515 nm, respectively.

To calculate the amount of eGFP molecules that were immobilized per vesicle, linear standards of each fusion protein (0–50 µg mL^−1^) at varying polymersome concentrations (0–0.5 % w/v) were prepared to account for absorbance and light scattering of the polymersomes at 485 and 515 nm and the resulting decrease in total fluorescence (Additional file 1: Figs. S2–S4).

### Confocal microscopy

Confocal microscopy images were taken on a FV-1000/IX81 confocal laser scanning microscope equipped with GaAsP detectors, an UPlanSApo ×60/1.20 objective and a 488 nm laser (Olympus, Tokyo, Japan). Exposure time was set to 2 µs/pixel, the laser was set to 500 *hv* with a gain of 1 and 3 % offset.

### Calcein leakage experiments

For leakage experiments, calcein (25 mM) was dissolved in 0.1 M Tris–HCl, pH 8.14 and the polymersomes were formed in the calcein solution according to the method described above. Polymersomes and free calcein were separated on a 140 mL SEC column packed with Sepharose 4B (GE Healthcare). Fifty microlitre of the purified polymersome dispersion were added to 50 μL of the protein solution in 96 well, chimney, black polystyrene assay plates (Greiner bio-one, Kremsmünster, Austria) immediately before fluorescence measurements. The sample fluorescence was measured for 12 h in an Infinite M200 microplate reader. Measurements were taken every 5 min with excitation and emission wavelengths of 485 and 515 nm, respectively. Negative controls were always included to differentiate between eGFP fluorescence and calcein fluorescence. The positive control included 3 % triton X-100 to disintegrate the polymersomes and force calcein release.

## Additional file

10.1186/s12951-016-0205-x Supplementary information on 1. Calcein leakage experiments in the presence of NP-40 and cholate,2. Cloning of the MBP-TEV-PolyAL-eGFP fusion protein, 3. Correction of eGFP fluorescence in presence of polymersomes including linear standards, 4. Molecular cloning of the fusion proteins.

## Abbreviations

PMOXA: poly(2-methyloxazoline)
PDMS: poly(dimethylsiloxane)
CecA: cecropin A
Cyt*b*_*5*_′: hydrophobic anchor of cytochrome *b*_*5*_
L′: hydrophobic anchor of lysis protein L
Vam3p′: hydrophobic anchor of syntaxin VAM3
PolyAL: poly(alanine-leucine)
NP-40: nonident P-40
