# Supplementary material for: Simple surface functionalization of polymersomes using non-antibacterial peptide anchors
Source: J Nanobiotechnology. 2016 Jun 22;14:48. doi: 10.1186/s12951-016-0205-x (PMC4918069; doi:10.1186/s12951-016-0205-x)
Supplement: Supplementary file 1 — 10.1186/s12951-016-0205-x Supplementary information on 1. Calcein leakage experiments in the presence of NP-40 and cholate,2. Cloning of the MBP-TEV-PolyAL-eGFP fusion protein, 3. Correction of eGFP fluorescence in presence of polymersomes including linear standards, 4. Molecular cloning of the fusion proteins. [file 12951_2016_205_MOESM1_ESM.docx]

**Simple surface functionalization of polymersomes using non-antibacterial peptide anchors**

**Ludwig Klermund, Sarah Poschenrieder, Kathrin Castiglione**

**Supporting information**

1. **Calcein leakage experiments in the presence of NP-40 and cholate**

Membrane integrity was investigated under various conditions including 0.75x and 1.5x the critical micellar concentration (CMC) of the surfactants nonident P-40 (NP-40) and cholate (Fig. S4). The polymersomes in the positive control were disintegrated with 3 % triton X-100. Especially NP-40 showed a strong increase in fluorescence and therefore a release of calcein into the aqueous surrounding. Above the CMC, approximately 36 % of the calcein was released by NP-40 within 12 h. Below the CMC, approximately 11 % calcein was released. Cholate had only minor effects on calcein release, with approximately 3 % calcein released above and below the CMC within 12 h.


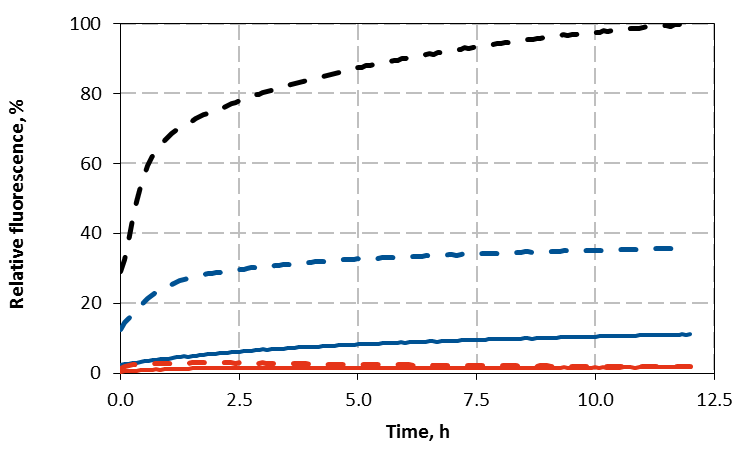


**Fig. S1** – Membrane integrity of calcein-loaded polymersomes in the presence of 0.75x (solid) and 1.5x (dashed) the critical micellar concentration (CMC) of NP-40 (blue) and 0.75 (solid) and 1.5x (dashed) the CMC of cholate (red). Treatment with 3 % triton X-100 is shown in dashed black.

1. **Cloning of MBP-TEV-PolyAL-eGFP**

The MBP-TEV-PolyAL-eGFP fusion protein was cloned using standard cloning procedures. The PolyAL-eGFP construct was amplified from pET21a(+)-PolyAL-eGFP with primers 5'-GATACACCATGGGCGAACATGCTCGCAG-3' and 5’-GATACAGGTACCCTTGTACAGCTCGTCCATG-3’ and digested with the restriction enzymes NcoI and KpnI. The digested gene was cloned into the pETM41 vector (European Molecular Biology Laboratory, Heidelberg, Germany) containing an N-terminal MBP moiety upstream of a TEV protease cleavage site. The plasmid was transformed into *E. coli* DH5α (Invitrogen, Carlsbad, USA) for plasmid propagation and into *E. coli* BL21 (DE3) (Novagen, Madison, USA), *E. coli* C41 (DE3) or *E. coli* C43 (DE3) ((Lucigen, Middleton, USA) for protein expression.

1. **Correction of eGFP fluorescence in presence of polymersomes**

The amount of immobilized eGFP was calculated from the effective fluorescence intensity *fl* of functionalized polymersomes. We observed inner filter effects during eGFP measurements caused by light absorption and light scattering of the polymersomes at the excitation (485 nm) and emission (515 nm) wavelengths of eGFP. This ultimately led to a reduction of eGFP fluorescence with increasing polymersome concentration (Fig. S1). To account for these inner filter effects when measuring eGFP fluorescence, eight standard curves of each eGFP fusion protein were analyzed at 0 – 0.5 % w/v polymersome concentration, resulting in eight straight lines of the form

| $fl=a_{i} \cdot c_{eGFP}$ | Eq. S1 |
| --- | --- |

with eight slopes, *a*_0_ – *a*_7_, per fusion protein. For each fusion protein, the slopes *a*_0_ through *a*_7_ decreased with increasing polymersome concentration, leading to the expected reduction in fluorescence in the presence of polymersomes.

Slopes *a*_0_ – *a*_7_ were plotted against the respective polymersome concentration (Fig. S2, exemplarily shown for eGFP without peptide anchor) to obtain a straight line of the form

| $a_{i}= a^{'}\cdot c_{polymersomes}+a_{0}$ | Eq. S2 |
| --- | --- |

with a slope *a’* and a y-intercept at the slope *a*_0_ (slope *a* at 0 % w/v polymersomes). After purification of eGFP-functionalized polymersomes from free eGFP, the polymersome concentration in each fraction was determined photometrically by measuring the absorbance at 350 nm. Fig. S3 shows that the absorbance of polymersomes at 350 nm was not significantly altered when adding 0 – 50 µg mL^-1^ eGFP. Subsequently, the eGFP concentration in each fraction was calculated from the effective fluorescence *fl* and the slope *a* at the given polymersome concentration according to:

| $c_{eGFP}= \frac{fl}{a}=\frac{fl}{a' \cdot c_{polymersome}+ a_{0}}$ | Eq. S3 |
| --- | --- |

Thus, the standard curves allowed for the determination of the correct eGFP concentration at a known polymersome concentration between 0 – 0.5 % polymersomes and the respective fluorescence intensity.


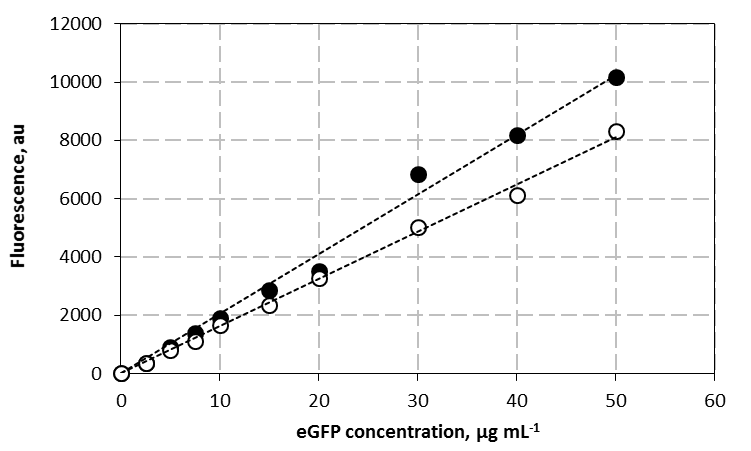


**Fig. S2** – Increase in fluorescence with increasing eGFP concentration with no polymersomes (black) and with 0.5 % w/v polymersomes (white).


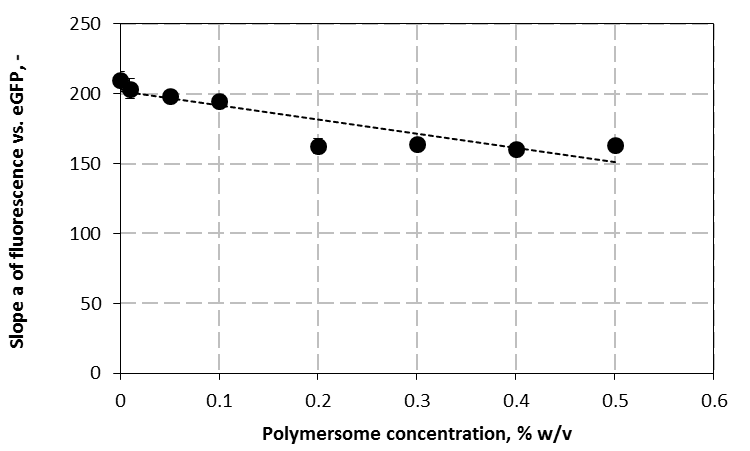


**Fig. S3** – Slopes of eGFP fluorescence with increasing eGFP concentration against polymersome concentration, resulting in a linear decrease of the fluorescence slope with increasing polymersome concentration.


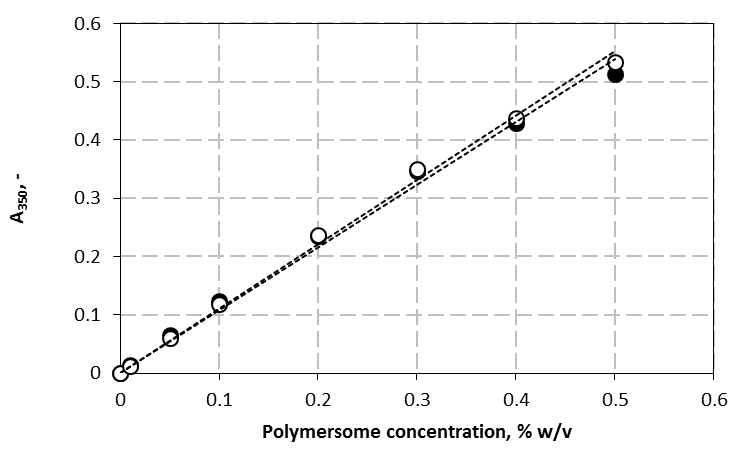


**Fig. S4** – Influence of eGFP concentration on the light extinction of the polymersomes at 350 nm. No change in light extinction of the polymersomes was measured when adding 0 µg mL^-1^ (black) and 50 µg mL^-1^ eGFP (white).

1. **Molecular cloning of the fusion proteins**

The nucleotide sequences of the peptide anchors are listed in Table S1. The gene fragment *cytb_5_’* was synthesized by biomers.net (Ulm, Germany). The gene fragments *vam3p’* and *polyAL* were synthesized by Eurofins Genomics (Ebersberg, Germany). The gene fragments *l’* and c*eca* were assembled via assembly PCR according to the Assembly PCR Oligo Maker (<http://www.yorku.ca/pjohnson/>). *Egfp* was amplified and digested with NdeI and EcoRI or EcoRI and XhoI for ligation into a linearized pET28a(+) and pET21a(+) vector (Novagen, Madison, USA), respectively. *Cytb_5_’*, *l’* and *vam3p’* were digested with EcoRI and XhoI and cloned into linearized pET28a(+)-eGFP, *ceca* and *polyAL* were amplified and digested with NdeI and EcoRI and cloned into linearized pET21a(+)-eGFP. A decaalanine linker was cloned between eGFP and each respective anchor via EcoRI. *E. coli* DH5α (Invitrogen, Carlsbad, USA) were transformed with each vector for selection and plasmid propagation.

**Table S1** – Nucleotide sequences of the peptide anchors

| **Peptide** | **Nucleotide sequence** |
| --- | --- |
| **Cyt*b_5_*’** | CTG AGC AAA CCG ATG GAA ACC CTG ATT ACC ACC GTG GAT AGC AAT AGC AGC TGG TGG ACC AAT TGG GTG ATT CCG GCG ATT AGC GCG CTG ATT GTG GCG CTG ATG TAT CGT CTG TAT ATG GCG GAT GAT |
| **L’** | CCA TTC AAA CAT GAG GAT TAC CCA TGT CGA AGA CAA CAA AGA AGT TCA ACT CTT TAT GTA TTG ATC TTC CTC GCG ATC TTT CTC TCG AAA TTT ACC AAT CAA TTG CTT CTG TCG CTA CTG GAA GCG GTG ATC CGC ACA GTG ACG ACT TTA CAG CAA TTG CTT ACT |
| **Vam3p’** | GTG ACC CTG ATT ATT ATT ATT GTG GTG TGC ATG GTG GTG CTG CTG GCG GTG CTG AGC |
| **PolyAL** | ATG GCG AAT ATG CTG GCA GCG TTG TTG GCA CTG TTG GCG GCA TTG CTG GCA TTG TTG GCG GCA TTG CTG GCA CTG TTG GCG GCA CTG CTG GCG |
| **CecA** | ATG AAA TGG AAG TTA TTT AAA AAG ATA GAA AAA GTT GGT CAG AAT ATT AGA GAT GGT ATA ATC AAA GCT GGA CCA GCT GTT GCA GTA GTA GGG GGA GCA ACA CAA ATT GCA AAA |
